# The healthcare resource impact of maternal mental illness on children and adolescents: UK retrospective cohort study

**DOI:** 10.1192/bjp.2021.65

**Published:** 2021-09

**Authors:** Holly Hope, Cemre Su Osam, Evangelos Kontopantelis, Sian Hughes, Luke Munford, Darren M. Ashcroft, Matthias Pierce, Kathryn M. Abel

**Affiliations:** 1 Centre for Women's Mental Health, Division of Psychology and Mental Health, Faculty of Biology, Medicine and Health, University of Manchester, UK; 2 Division of Informatics, Faculty of Biology, Medicine and Health, University of Manchester, UK; 3 Centre for Women's Mental Health, Division of Psychology and Mental Health, Faculty of Biology, Medicine and Health, University of Manchester, UK; and Population Health Analysis, Department of Health and Social Care, UK; 4 Division of Population Health, Health Services Research & Primary Care, Faculty of Biology, Medicine and Health, University of Manchester, UK; 5 Centre for Pharmacoepidemiology and Drug Safety, Faculty of Biology, Medicine and Health, University of Manchester, UK; 6 Centre for Women's Mental Health, Division of Psychology and Mental Health, Faculty of Biology, Medicine and Health, University of Manchester, UK; and Greater Manchester Mental Health NHS Foundation Trust, UK

**Keywords:** Depressive disorders, psychotic disorders, primary care, in-patient treatment, out-patient treatment

## Abstract

**Background:**

The general health of children of parents with mental illness is overlooked.

**Aims:**

To quantify the difference in healthcare use of children exposed and unexposed to maternal mental illness (MMI).

**Method:**

This was a retrospective cohort study of children aged 0–17 years, from 1 April 2007 to 31 July 2017, using a primary care register (Clinical Practice Research Datalink) linked to Hospital Episodes Statistics. MMI included non-affective/affective psychosis and mood, anxiety, addiction, eating and personality disorders. Healthcare use included prescriptions, primary care and secondary care contacts; inflation adjusted costs were applied. The rate and cost was calculated and compared for children exposed and unexposed to MMI using negative binomial regression models. The total annual cost to NHS England of children with MMI was estimated.

**Results:**

The study included 489 255 children: 238 106 (48.7%) girls, 112 741 children (23.0%) exposed to MMI. Compared to unexposed children, exposed children had a higher rate of healthcare use (rate ratio 1.27, 95% CI 1.26–1.28), averaging 2.21 extra contacts per exposed child per year (95% CI 2.14–2.29). Increased healthcare use among exposed children occurred in inpatients (rate ratio 1.37, 95% CI 1.32–1.42), emergency care visits (rate ratio 1.34, 95% CI 1.33–1.36), outpatients (rate ratio 1.30, 95% CI 1.28–1.32), prescriptions (rate ratio 1.28, 95% CI 1.26–1.30) and primary care consultations (rate ratio 1.24, 95% CI 1.23–1.25). This costs NHS England an additional £656 million (95% CI £619–£692 million), annually.

**Conclusions:**

Children of mentally ill mothers are a health vulnerable group for whom targeted intervention may create benefit for individuals, families, as well as limited NHS resources.

## Background

Much attention has been paid to the risk of mental illness in children and adolescents exposed to parental mental illness, but it is increasingly clear that the overall health (including physical health) of these young people requires further investigation.^1^ This is particularly important given the large numbers of children and young people with parental mental illness,^2^ and the fact that, unlike mental illness^3^ and death,^4^ physical health problems are common in childhood.^1^ Understanding the potential additional resource use and costs associated with caring for the health of these easily identifiable children is vital for policy makers and healthcare commissioners to plan services that meet their needs.^5^

Previous work, including our recent studies,^1,6,7^ suggests that mothers with mental illness are less likely to take up preventive public health services,^8–11^ including childhood vaccinations,^7,8,12,13^ and may be more likely to use acute health services^6,12,14–17^ for their children than mothers without mental illness. These analyses focus on maternal depression^9,12,13,17^ and secondary care outcomes.^14–17^ For example, in a UK registry study (*N* = 25 252), children of mothers with depression used in-patient and emergency services significantly more often than children of mothers without depression (odds ratio 1.67, 95% CI 1.32–2.11).^17^

## This analysis

The aim of our programme of work with children and adolescents exposed to parental mental illness is to describe the broader needs of these children; in England, we confine our sample to children living with maternal mental illness (MMI). Few studies have examined healthcare use by children of ill parents across mental illness categories, or examined differences by age group, and there is no information from UK sources about the current financial costs associated with this. Here, we quantify the differences in healthcare use associated with MMI and the direct medical costs. We first hypothesised that any MMI would increase use of healthcare resources and significantly increase costs in exposed children; our second hypothesis was that children exposed to serious MMI would use acute resources more, but other non-acute care less, than comparator children.

## Method

### Data

This retrospective cohort study used linked primary and secondary care electronic health records to investigate healthcare use of children aged 0–17 years, over a 10-year period between 1 April 2007 and 31 July 2017.

The National Health Service (NHS) is England's publicly funded healthcare system, and most of its budget is distributed to approximately 200 clinical commissioning groups who fund local primary, secondary and emergency care, among other services. All services are free at the point of access, and prioritise on clinical need. Over 98% of people in England are registered with a doctor (general practitioner; GP) at a single general practice, where they receive primary care and prescriptions, which is similar to care received from family physicians in the USA. GPs will refer patients to secondary healthcare settings (e.g. general and specialist hospital care, mental health and community services) for further investigation or more advanced therapies; most private healthcare providers require a GP referral before commencing treatment. Separate to this are accident and emergency care (A&E) centres for life-threatening illnesses or accidents, which require immediate, intensive treatment and no referral from a GP.

Data on the cohort, exposure and part of the outcome data are from the Clinical Practice Research Datalink (CPRD GOLD). The CPRD is a primary care database of approximately 15.3 million patients (broadly representative of the UK population). It includes data about GP clinical consultations, prescriptions (including those issued in secondary care) and referrals to secondary care services.

The study cohort was drawn from children identified in the CPRD GOLD ‘mother–baby’ link. The mother–baby link matches children to their mothers by using a family identifier (based on residential address) and an algorithm that matches delivery and birth records. Children were linked to their Hospital Episodes Statistics (HES) data via a unique patient identifier separate from the family identifier. HES captures data on every visit to a secondary healthcare provider funded by NHS England, including visits for private treatment. The HES data-sets are available for around 75% of CPRD GOLD practices in England, and capture visits that require a hospital bed (in-patient), appointments with specialist clinical services (out-patient) and emergency care visits (A&E). At extraction, HES-linked data were available from 1 April 2007 to 31 July 2017.

To calculate healthcare costs, tariffs were extracted from the following data-sets (2007–2017): the Health and Social Care annual calculation of primary care unit costs, which is a reference document that uses information on UK salaries, consultation times and other ancillary costs to calculate a unit cost per consultation; the NHS Digital Prescription Cost Analysis survey, which provides annual mean costs for drugs purchased by the NHS; and the NHS annual schedule of reference costs, which lists the average annual cost of an admission within secondary care settings (out-patient, hospital admission and A&E).

Adjustments for inflation between 2007–2017 were made with data from the consumer price index data-set, and child population data were taken from the Office of National Statistics (see Supplementary Appendix 1 available at https://doi.org/10.1192/bjp.2021.65 for a list of data-sets and key references).

### Ethical approval

The authors assert that all procedures contributing to this work comply with the ethical standards of the relevant national and institutional committees on human experimentation and with the Helsinki Declaration of 1975, as revised in 2008. The study was approved by the Independent Scientific Advisory Committee (ISAC) for Medicines and Healthcare products Regulatory Agency Database Research (protocol number 17_187). All observational research using CPRD patient data was approved by the NHS Health Research Authority's East Midlands – Derby Research Ethics Committee (reference number 05/MRE04/87).

### Study design and population

**Fig. 1:**
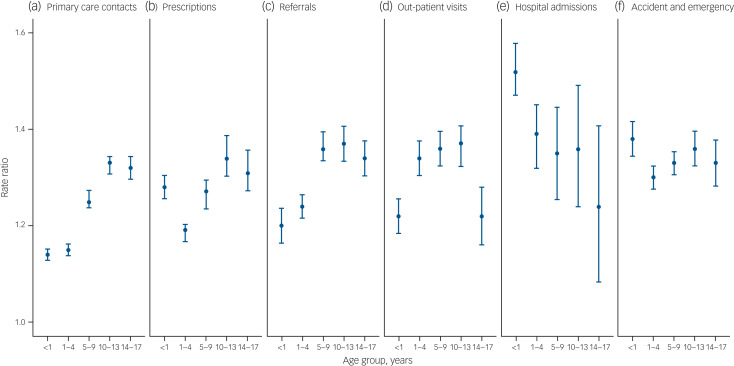
Rate ratios of (a) primary care contacts, (b) prescriptions, (c) referrals, (d) out-patient visits, (e) in-patient admissions and (f) accident and emergency care visits of children exposed to maternal mental illness compared with those who were not exposed to maternal mental illness.

Children identified in the mother–baby link were eligible if they were born between 1 January 1993 and 31 June 2017 and registered for at least 30.5 days (during age 0–17 years) at a CPRD-participating general practice in England between 1 April 2007 and 31 July 2017 (*N* = 707 698; Fig. 1). Children were excluded if there was no linkage to HES; their mother was not registered at an up-to-standard general practice at the child's start of follow-up; or their mother had <2 years data before or after the child's start of follow-up, to ascertain exposure to MMI.

Follow-up began either the child's date of birth, the date the practice began contributing CPRD up-to-standard data, the child's registration date or the study start date (1 April 2007), whichever came last. The follow-up period ended on the date the child transferred out of the practice, the child's 18th birthday, the child's date of death, the mother's date of death, the practice data collection end date or the study end date (31 July 2017), whichever came first. The child's date of birth was used to split the child's follow-up period into pre-defined age groups of infancy (<1 year), pre-school (1–4 years), primary school (5–9 years), middle school (10–13 years) and adolescence (14–17 years).

### Exposure

For each age group, children were classed as exposed if their mother had a mental illness between 2 years before the start of that age group and the end of that age group. MMI was defined using diagnoses symptoms and prescription data from the mother's primary care health record. MMI included the following illnesses: non-affective psychosis, affective psychosis, depression, anxiety, eating disorders, personality disorders and substance and alcohol misuse disorders.

Prescription and symptom codes were assigned to a mental illness diagnosis (e.g. antipsychotics assigned to non-affective psychotic disorder, anxiousness assigned to anxiety disorder). A case of mental illness included either a diagnosis, a prescription for a therapy ≤3 months of a symptom of the same mental illness or a prescription/symptom and a matching historical diagnosis (see Abel et al for further details^2^). For the purpose of this analysis, we created the following mental illness categories: common mental illness (depression or anxiety) serious mental illness (non-affective or affective psychotic disorder) and addiction disorders (substance and alcohol misuse disorders). Note that parental personality or eating disorders were not represented in these three subcategories; however, they were represented under ‘any’ parental mental illness. In addition, children could appear in more than one exposure group.

### Healthcare use

Healthcare use was investigated by type: primary care contacts, prescriptions, referrals, hospital admissions, out-patient visits and A&E visits. A primary care contact was defined as a face-to-face consultation, a home visit or telephone contact with a GP, nurse, midwife or healthcare visitor about a child. The numbers of referrals and prescriptions from primary care were counted. Multiple contacts/prescriptions of the same type, on the same date, were counted once.

### Healthcare costs

Primary care contacts were costed according to consultation (face to face, telephone contact, home visit) and staff type (doctor, nurse, healthcare visitor, other healthcare professional). Prescriptions were costed per prescription, and where specific costs were not provided by the NHS Digital Prescription Cost Analysis survey (29.5% of all prescriptions), the annual average unit cost was applied.

The average annual unit cost was applied to each A&E visit and out-patient admission. A hospital admission was costed by type of episode (day case, regular day/night case, elective or non-elective admission) and length of stay where unit cost (bed day) was dependent on it being an elective or non-elective admission. All unit costs were adjusted for inflation, with 2017 considered as the base year.

### Other variables

An area-level measure of deprivation was extracted with the 2010 quintile of the Index of Multiple Deprivation (IMD), based on residential address, which ranks areas in order of poverty, with the fifth quintile representing children living in the most deprived area. Child ethnicity data were captured from both the HES data-set and the CPRD, and categorised into Asian, Black, mixed, other and White.

### Statistical analyses

For each age group, MMI exposure and healthcare use outcome, the rate of healthcare events and associated costs were calculated by dividing the number of number of events by person-years of follow-up. Rates were compared between levels of MMI exposure, using negative binomial regression models with the log of the year's follow-up as an offset term to account for differential follow-up. These models provided two different comparisons: the rate ratio (rate exposed divided by rate unexposed) and rate difference (rate exposed minus the rate unexposed) measures of relative and absolute difference between groups. These models controlled for period in the analysis (the year the age group started as a categorical variable), and included an interaction term between age group and exposure, to calculate age group-specific associations. To account for clustering by maternal sibships, s.e. values were calculated with the robust Huber–White estimator, accounting for clustering by mother.

Cost differences were calculated for each age group as the difference in the rate of costs (in pounds per child per year) between exposed and unexposed children. The annual excess NHS spend in England associated with MMI was first estimated by multiplying age group-specific estimates for: the cost difference, the prevalence of MMI and the number of children in England. These were then summed over the age groups, to derive an estimate of the total excess annual spend associated with MMI. Confidence intervals for the cost differences and the annual excess NHS cost were estimated from 1000 bootstrap samples, using the normal approximation for the test statistic.

### Sensitivity analyses

Three sensitivity analyses were devised. In the first, we assessed if a link between healthcare use was evident after redefining MMI as ≤2 years before the start of each age group (i.e. ignoring exposure during that age group), to assess the role of reverse causation in this study. In the second, we considered whether the association between MMI and childhood healthcare use could be explained by area-level deprivation, and adjusted for IMD quintile. Finally, the selection of children with HES linkage had the potential to affect our analysis; to assess this, we compared the rates of primary care contacts for all eligible children without HES linkage (*n* = 630 351).

## Results

### Cohort description

The final analysis cohort contained 489 255 children (Supplementary Fig. 1). In the first year of follow-up, 112 741 (23.0%) children were exposed to MMI, of whom 54 517 (48.7%) were girls; the median maternal age at birth was 30 years (interquartile range 25–34) and the median follow-up was 5.34 years (interquartile range 2.85–7.71). Compared with unexposed children, children exposed to MMI were more likely to be from an area of England in the highest quintile of deprivation (19.7 *v.* 15.0%). Children from Black and Asian backgrounds were underrepresented in exposed compared with unexposed groups (Supplementary Table 1).

### Healthcare use

Children exposed to MMI accessed healthcare at a higher rate than children who were not exposed to MMI (rate ratio 1.27, 95% CI 1.26–1.28; rate difference 2.21, 95% CI 2.14–2.29; Table 1); these effects remained after adjusting for IMD quintile (rate ratio 1.26, 95% CI 1.24–1.27; rate difference 2.16, 95% CI 2.08–2.24; Supplementary Table 2). Sensitivity analyses produced similar results (Supplementary Tables 3 and 4).

**Table 1:** The total rate (per child per year) of healthcare use for children exposed and unexposed to maternal mental illness

			Age group, years				
Children's exposure to MMI		Overall^a^	<1	1–4	5–9	10–13	14–17
Healthy mothers	*n*	814 306	200 310	231 996	182 524	126 399	73 107
	Rate	9.56	18.3	9.46	5.62	5.17	6.38
Any MMI	*n*	295 276	47 711	86 497	76 080	52 573	32 865
	Rate	11.8	22.1	11.3	7.19	6.96	8.38
	Rate ratio (95% CI)	1.27 (1.26−1.28)	1.21 (1.20−1.22)	1.19 (1.18−1.20)	1.28 (1.26−1.30)	1.34 (1.32−1.37)	1.31 (1.29−1.34)
	Rate difference (95% CI)	2.21 (2.14−2.29)	3.80 (3.61−3.99)	1.80 (1.70−1.89)	1.57 (1.48−1.66)	1.78 (1.67−1.90)	2.00 (1.85−2.15)
Common MMI	*n*	292 281	47 008	85 500	75 243	52 015	32 515
	Rate	11.8	22.1	11.3	7.19	6.97	8.39
	Rate ratio (95% CI)	1.27 (1.26−1.28)	1.21 (1.20−1.22)	1.19 (1.18−1.20)	1.28 (1.26−1.30)	1.35 (1.32−1.37)	1.32 (1.29−1.34)
	Rate difference (95% CI)	2.23 (2.15−2.30)	3.83 (3.64−4.03)	1.80 (1.71−1.90)	1.58 (1.48−1.67)	1.80 (1.69−1.91)	2.01 (1.86−2.17)
Serious MMI	*n*	5553	787	1520	1469	1079	698
	Rate	12.0	20.5	11.5	8.38	7.84	9.83
	Rate ratio (95% CI)	1.37 (1.31–1.43)	1.12 (1.05−1.20)	1.22 (1.16−1.29)	1.49 (1.38−1.61)	1.52 (1.39−1.65)	1.54 (1.40−1.69)
	Rate difference (95% CI)	2.48 (1.97–3.00)	2.21 (0.89−3.53)	2.08 (1.45−2.71)	2.76 (2.14−3.38)	2.67 (2.01−3.33)	3.45 (2.52−4.38)
Addiction disorders	*n*	7140	875	1782	1977	1500	1006
	Rate	11.5	21.5	10.6	7.11	6.27	7.28
	Rate ratio (95% CI)	1.18 (1.14−1.23)	1.20 (1.13−1.27)	1.12 (1.07−1.18)	1.19 (1.15−1.33)	1.18 (1.10−1.29)	1.18 (1.07−1.29)
	Rate difference (95% CI)	1.78 (1.32−2.24)	3.61 (2.35−4.87)	1.18 (0.62−1.74)	1.34 (0.84−1.84)	0.98 (0.49−1.79)	1.12 (0.44−1.79)

MMI, maternal mental illness.

a. Refers to total number of child observations; children appear in more than one age group.

The rate ratio of healthcare use was highest for older children (age group 14−17 years: rate ratio 1.31, 95% CI 1.29–1.34; age group <1 year 1.21, 95% CI 1.20–1.22). Conversely, the largest rate difference estimates were observed for infants: exposed children aged <1 year used almost four more healthcare units per year than unexposed children (rate difference 3.80, 95%CI 3.61–3.99), whereas children aged 14–17 years used two more healthcare units per year than unexposed children (rate difference 2.00, 95% CI 1.85–2.15).

The rate ratio for serious MMI was largest at older age groups (age group <1 year: rate ratio 1.12, 95% CI 1.05–1.20; age group 14–17 years: rate ratio 1.54, 95% CI 1.40–1.69). There was a similar, less-marked difference by age for common maternal illness (age group <1 year: rate ratio 1.21, 95% CI 1.20–1.22; age group 14−17 years: rate ratio 1.32, 95% CI 1.29–1.34). By contrast, the relative healthcare use of children exposed to addiction disorders did not vary by age (age group <1 year: rate ratio 1.18, 95% CI 1.14–1.23; age group 14−17 years: rate ratio 1.18, 95% CI 1.07–1.29).

### Primary care

**Fig. 2:**
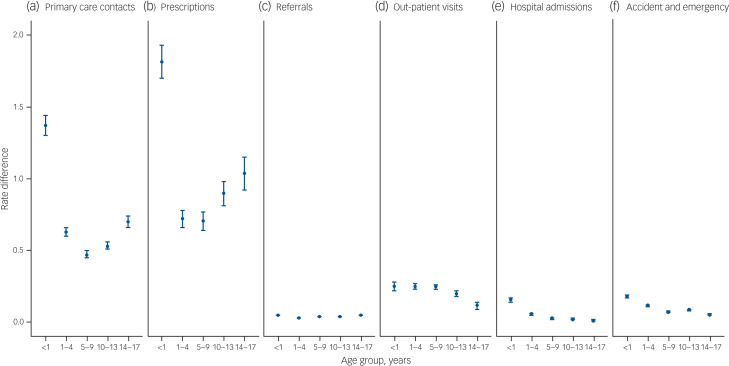
Rate differences of (a) primary care contacts, (b) prescriptions, (c) referrals, (d) out-patient visits, (e) in-patient admissions and (f) accident and emergency care visits of children exposed to maternal mental illness compared with those who were not exposed to maternal mental illness.

Children exposed to MMI had more primary care contacts than unexposed children (5.02 *v.* 4.27 contacts per child per year; rate ratio 1.24, 95% CI 1.23–1.25; rate difference 0.75; 95% CI 0.73–0.78), these effects were evident across all age-groups (Figs. 1 and 2). Only children aged under 1 of mothers with serious mental illness did not have significantly different rates of primary care use (rate ratio 1.04, 95% CI 0.99–1.09; Supplementary Table 5, estimates are tabulated).

### Prescriptions

Children exposed to any MMI were administered prescriptions at a higher rate than unexposed children (4.92 *v.* 3.90 prescriptions per year; rate ratio 1.28, 95% CI 1.26–1.29), equivalent to 1.02 additional medications per child per year (95% CI 0.97–1.07). Children exposed to serious MMI were prescribed more medications at all ages (rate ratio 1.42, 95% CI 1.33–1.51), particularly at age 14–17 years (5.36 *v.* 3.30; rate ratio 1.63, 95% CI 1.43–1.85; rate difference 2.06, 95% CI 1.37–2.75). The types of drugs more often prescribed to exposed children included those used to treat mental and behavioural (rate ratio 1.77, 95% CI 1.76–1.79), gastrointestinal (rate ratio 1.54, 95% CI 1.53−1.55), musculoskeletal (rate ratio 1.45, 95% CI 1.44−1.47), gynaecological and urinary tract (rate ratio 1.41, 95% CI 1.39−1.43), and respiratory (rate ratio 1.35, 95% CI 1.34−1.36) diseases, as well as infections (rate ratio 1.25, 95% CI 1.25–1.26) (Supplementary Table 6).

### Referrals

There was a 30% (95% CI 29% to 31%) increase in the rate of referrals to secondary care that were associated with MMI. Children exposed to serious MMI experienced the highest relative increase (rate ratio 1.41, 95% CI 1.33–1.50), particularly at age 5–9 years (rate ratio 1.64, 95%CI 1.48–1.81). The smallest exposed/unexposed increment came from alcohol and substance misuse (rate ratio 1.16, 95% CI 1.10–1.23).

### Out-patient visits

Children exposed to MMI visited out-patient clinics more frequently than unexposed children (0.99 *v.* 0.76; rate ratio 1.30, 95% CI 1.28–1.32), which is equivalent to 0.23 (95% CI 0.22–0.24) additional visits per child per year. The relative increase was greatest for serious MMI (rate ratio 1.41, 95% CI 1.30–1.53; rate difference 0.319, 95% CI 0.22–0.39).

### In-patient admissions

There was a 37% (95% CI 32–42%) increase in the rate of in-patient admissions for children exposed to any MMI (0.21 *v.* 0.14 admissions per child per year). The association between common MMI and rate of hospital admissions attenuated with age: the highest relative and absolute rate differences were at age <1 year (rate ratio 1.52, 95% CI 1.48–1.57; rate difference 0.16, 95% CI 0.14−0.17), and the lowest were at age 14–17 years (rate ratio 1.24, 95% CI 1.11–1.39; rate difference 0.01, 95% CI 0.01–0.02).

### A&E visits

The rate of A&E visits was 34% (95% CI 32–35%) higher for children exposed to any MMI. The highest increase in A&E visits was greatest at age <1 year for children exposed to common MMI (0.65 *v.* 0.47 visits; rate ratio 1.38, 95% CI 1.35–1.41), and at age 5–9 years for children exposed to serious MMI (0.33 *v.* 0.23 visits; rate ratio 1.46, 95% CI 1.33–1.60).

### Healthcare costs

The cost of healthcare per child per year for children varied according to the age of the child and exposure to MMI (Supplementary Table 6). The first year of life was the most costly: children exposed to MMI cost £3076 per child per year compared with £2211 for unexposed children, meaning a cost difference of £864 (95% CI £810–£918) per exposed child per year. Excess healthcare costs reduced with child age: at age 14–17 years, the cost per exposed child per year was £618 compared with £494 per unexposed child per year (cost difference £124, 95% CI £104–£143), which is evidence of a smaller but still substantial additional spend associated with MMI. In-patient admissions and out-patient visits are cost-intensive, and explain most of the excess healthcare costs (Supplementary Table 7). We estimate the excess cost to NHS England associated with children being exposed to MMI is £655 645 000 (95% CI £618 916 000–£692 373 000; Table 2), annually.

**Table 2:** The additional annual cost to the NHS in England of children exposed to maternal mental illness

Costs per age group	Exposed to MMI	Not exposed to MMI	A: Cost difference (95% CI)	B: ONS population data^a^	C: % with MMI	NHS extra spend (A×B×C)
<1 year	£3076	£2211	£864 (£810−£918)	696 441	19.2	£115 759 981
1–4 years	£1128	£875	£253 (£234−£272)	2 696 915	27.2	£185 548 467
5–9 years	£764	£616	£148 (£133−£163)	3 083 582	29.4	£134 482 975
10–13 years	£745	£572	£174 (£156−£192)	2 379 741	29.4	£121 378 183
14–17 years	£618	£494	£124 (£104−£143)	2 566 631	31.0	£98 475 206
Total						£655 645 000 (95% CI £618 916 000−£692 373 000)

NHS, National Health Service; MMI, maternal mental illness; ONS, Office of National Statistics.

a. Based on 2016 estimates.

## Discussion

### Summary of main findings

In this analysis, we describe and quantify the excess in child healthcare use associated with exposure to MMI, and estimate excess costs associated with this healthcare use. As hypothesised, MMI was associated with increased child healthcare use in primary and secondary care, and this excess was estimated to cost NHS England an extra £656 million, annually. The majority of the excess costs arise from hospital admissions and occur in the first year of life, although significant excess healthcare use persists throughout childhood. There was limited support for the hypothesis that children exposed to serious MMI would use more acute and less non-acute healthcare resources than unexposed children and children exposed to common MMI. Instead, these children presented more frequently across all healthcare settings. Prescription data indicate that, compared with unexposed children, children living with MMI receive more drug prescriptions, in particular for mental and behavioural disorders, but also for physical, acute and chronic diseases. Although we cannot infer causality from these findings, the association between MMI and child healthcare use was independent of socioeconomic status.

### Research in context

Our results are consistent with previous studies that investigated healthcare use among children exposed to parental mental illness. Sills et al^9^ (*N* = 69 655 children aged 0–17 years, living in the USA) reported similar increases in visits to primary, secondary and acute healthcare, but not preventive primary care associated with MMI; they also reported that healthcare use varied by age, with the highest use among the youngest children. Our effect of common MMI on A&E visits at 1–4 years of age (rate ratio 1.30) is comparable with the odds of an A&E admission at 30–33 months (odds ratio 1.44) associated with maternal depression that was reported by Minkovitz et al.^12^ The effect of serious MMI on in-patient admissions (rate ratio 1.38) is higher than the incidence rate ratio of 1.17 for hospital admissions that was reported by Ranning et al, who used a large Danish register (*N* = 2 000 694).^16^ However, Ranning et al followed offspring until 30 years of age (a different approach) and included paternal mental illness, which exerts less influence on children's healthcare outcomes.^1^

Women with serious mental illness are perceived to be high-risk mothers, and are more likely to have their children placed under social services supervision or permanently removed from their care, compared with healthy mothers or those with depression or anxiety disorders.^10,18^ Therefore, we anticipated children exposed to serious MMI would use acute healthcare more frequently than unexposed children and children exposed to common MMIs. Our findings do not examine the reasons for A&E visits or in-patient admissions, but, apart from age 5–9 years, we did not find that serious MMI was associated with more healthcare use, and child contacts with acute services were similar across MMI groups.

Using Swedish registers, we recently demonstrated that children are at greater risk of injury when exposed to maternal common or addiction disorders compared with serious MMI.^6^ These parallel findings suggest two potential phenomena that warrant further investigation: first, that mothers with serious mental illness pose no greater risk to their children than mothers with other mental illnesses; and second, that mothers with more severe mental illness fail to access acute services when needed, for fear their child may be taken into care.^26^

### Strengths and limitations

This study uses linked population-based hospital and primary care electronic health records to calculate the healthcare resource use of children in England (see Supplementary Appendix 1 for more information). Utilising health registers reduces some of the inherent biases that occur in prospective cohort studies, such as small sample sizes, loss to follow-up and selection bias. Linkage from primary care to secondary care data-sets require GP practice consent. Therefore, analysis of those with secondary care data may introduce a possible selection bias. However, when we repeated our analysis of primary care outcomes in those without consent for secondary care linkage, we observed almost equal relative and absolute effect sizes (Supplementary Table 4).

There are several limitations. First, we were unable to address the effect of paternal mental illness on child healthcare use. Second, we did not test whether this association was independent of other covariates, such as maternal age, lifestyle factors (e.g. smoking), education and employment.^19^ This was because the aim was to describe the health inequality and costs associated with MMI as a target for clinical and policy intervention, rather than assess whether this had a causal connection. However, when we did adjust for a measure of socioeconomic deprivation, we found similar sized effects. Medical records lack data on employment, income and housing, and so we were unable to test the independence of MMI from these and other indicators of poverty. Third, the prescription rate is a measure of prescriptions administered rather than prescriptions claimed, and so consequent costs of medication use may be overestimated. Fourth, unit costs are averages of adult and paediatric costs, and do not reflect the high costs attached to rare, resource-intensive diseases of childhood such as cancer and cystic fibrosis. Fourth, use of child and adolescent mental health services is incompletely recorded in HES and, given that there is a risk of neurodevelopmental, emotional and behavioural problems among children exposed to MMI, the healthcare use increments associated with MMI may be underestimates. Fifth, HES data misses healthcare that occurs at non-NHS sites; however, we anticipate that this will include a very small number of children, and so do not see this as a major source of bias. Sixth, we have only accounted for the direct healthcare costs of these children; the personal costs to their quality of life and the societal costs associated with this increased healthcare use (e.g. days of work lost by the parent) are unaccounted for. Every healthcare contact from 5 years onward represents at least 1 day outside of education; therefore, these findings may partly explain prior analyses that demonstrate an association between parental mental health and poorer educational attainment in offspring.^20^

### Possible explanatory mechanisms of increased healthcare use

It is tempting to conclude that the high frequency of healthcare use and excess prescriptions among children exposed to MMI may be explained by inappropriate use of healthcare by ill mothers. This might include a mother accessing acute care services (such as A&E) for non-acute reasons and/or poor uptake of healthy baby checks and preventive interventions such as vaccinations; both should result in increased use of acute services and lower use of primary care. Instead, we report increases in acute and primary care visits among exposed children. Moreover, if exposed children were presenting unnecessarily, we should observe a higher rate of primary care consultations, but a similar rate of medications, referrals and use of clinical specialists. However, children exposed to MMI had more frequent prescriptions, referrals and visits to out-patient specialist care. This suggests that although some increase in healthcare services might relate to maternal health anxiety, it is also likely to be driven by genuinely greater healthcare need among these children. In our view, this interpretation is more consistent with our recent finding in Sweden and our systematic review of physical health in children and adolescents exposed to parental mental illness.^1^ The circumstances that underpin MMI also contribute to excess healthcare use among offspring. The association between MMI and increased rates of prescriptions indicated for neurodevelopmental, autoimmune and inflammatory disorders among offspring suggest the possibility of shared genetic and environmental risks that combine to create multi-morbid families.^21^

### Future research

It is possible that common childhood health problems, such as asthma, atopy, obesity and tooth decay, are drivers of the health inequality associated with MMI.^1^ Although we can make some inferences about the reasons for excess healthcare use from the pattern of healthcare use and type of drug prescribed, future research should further isolate the specific types of health needs that are overrepresented in these children, and how parental mental illness and offspring morbidity cluster. This may also provide insights into the shared genetic and environmental factors that underpin these associations. Future research may also give more detail about how excess cost is distributed across different groups; for example, regionally or by ethnic group. Finally, quantifying the contribution of family adversity, lifestyle factors (e.g. maternal smoking), comorbid maternal physical illness and obesity to the excess healthcare use among children with mentally ill mothers^19^ will provide important information on causal mechanisms and identify crucial points of intervention.

### Clinical implications

Identifying sources of maternal resilience and leveraging them to maintain well-being is likely to be key for interventions to improve the health and lives of children and adolescents exposed to parental mental illness. These might include maternal health literacy and supportive social networks. As most women with a mental illness have children, increasing awareness (through training) in primary care professionals of the broader implications of maternal mental health is important. This would mean offering reproductive health and family planning to women, with tailored information about modifiable risks, such as smoking cessation and drug, alcohol and medication use during pregnancy. Evidence suggests that people with mental illness are less able to take advantage of public health campaigns.^11,22^ Mothers with mental illness and their children require interventions that are cognisant of their needs. Although fear and stigma may prevent a parent with mental illness from accessing timely help for their child's chronic health conditions, simple measures addressing factors like access to transport are relatively easy to implement. More complex, highly relevant factors might be more important, but more challenging to address (e.g. intimate partner violence or socioeconomic deprivation).^23,24^ Primary care and other professionals should be encouraged to ask about these in a safe setting, to monitor them and to understand ways in which they influence a mother's ability to access healthcare for herself and her children.

### Policy implications

The additional annual cost attached to these children (£656 million) represents <1% of the annual £115 billion NHS budget, but it is £100 million more than the total budget deficit that the overall NHS provider sector forecasted for 2019.^25^ In a prior analysis, we suggested that the number of children exposed to MMI in the UK may be increasing,^2^ and so this excess cost is also likely to increase. Our analysis ends at 17 years of age, but we hypothesise that the excess burden to healthcare systems and an individual's poorer health continues throughout adulthood. Policies such as the healthy child programme^26^ could target children of mentally ill mothers, who clearly represent an group with unmet needs. Interventions that target the largest absolute healthcare use increment associated with MMI in the first year of life are likely to differ from strategies that aim to reduce the largest relative increase in healthcare use observed at older ages, where schools may play an important role in protecting the well-being of their students and preventing early multi-morbidity among children and adolescents exposed to parental mental illness.

The broader health needs of children exposed to MMI (and their mothers) are overlooked. We demonstrate that there is a health vulnerability attached to children exposed to MMI evident across both the poorest and the wealthiest areas of England. This research presents the excess healthcare use and cost of children exposed to MMI, and identifies further avenues for research that may help these families and reduce the burden on the NHS.

## Data Availability

Read codes used are published on Clinicalcodes.org. Electronic health records are, by definition, considered ‘sensitive’ data in the UK by the Data Protection Act 2018, and cannot be shared via public deposition because of information governance restriction in place to protect patient confidentiality. Access to data are available only once approval has been obtained through the individual constituent entities controlling access to the data. The primary care data can be requested via application to the Clinical Practice Research Datalink (www.cprd.com/researcher); secondary care data can be requested via application to the Hospital Episode Statistics from the UK Health and Social Care Information Centre (www.hscic.gov.uk/hesdata).

## Supplementary material

For supplementary material accompanying this paper visit https://doi.org/10.1192/bjp.2021.65. click here to view supplementary material

## References

[1] Pierce M, Hope H, Kolade A, Gellatly J, Osam CS, Perchard R, Effects of parental mental illness on children's physical health: systematic review and meta-analysis. Br J Psychiatry 2020; 217: 354–63. 31610824 10.1192/bjp.2019.216

[2] Abel KM, Hope H, Swift E, Parisi R, Ashcroft DM, Kosidou K, Prevalence of maternal mental illness among children and adolescents in the UK between 2005 and 2017: a national retrospective cohort analysis. Lancet Public Health 2019; 4(6): e291–300. 31155222 10.1016/S2468-2667(19)30059-3 PMC6557735

[3] Dean K, Green MJ, Laurens KR, Kariuki M, Tzoumakis S, Sprague T, The impact of parental mental illness across the full diagnostic spectrum on externalising and internalising vulnerabilities in young offspring. Psychol Med 2018; 48(13): 2257–63. 29331151 10.1017/S0033291717003786

[4] Webb R, Abel KM, Pickles AR, Appleby L, King-Hele SA, Mortenson PB. Mortality risk among offspring of psychiatric inpatients: a population-based follow-up to early adulthood. Am J Psychiatry 2006; 163(12): 2170–7. 17151170 10.1176/appi.ajp.163.12.2170

[5] Abel KM, Morgan VA. Women and mothers with mental illness, and their children. In Textbook in Psychiatric Epidemiology (3rd edn) (eds MT Tsuang, M Cohen, PB Jones): 483–515. John Wiley & Sons, 2011.

[6] Nevriana A, Pierce M, Dalman C, Wicks S, Hasselberg M, Hope H, Maternal and paternal mental illness and risk of injuries in children and adolescents: a nationwide register-based study in Sweden. BMJ 2020; 369: m853. 32269017 10.1136/bmj.m853 PMC7190076

[7] Osam CS, Matthias P, Hope H, Ashcroft DM, Abel KM. The influence of maternal mental illness on vaccination uptake in children: a UK population-based cohort study. Eur J Epidemiol 2020; 35: 879–89. 32328992 10.1007/s10654-020-00632-5 PMC7524844

[8] Davidsen KA, Christiansen E, Haubek D, Asmussen J, Ranning A, Thorup AAE, Parental mental illness, attendance at preventive child healthcare and dental caries in the offspring: a nation-wide population-based cohort study. Soc Psychiatry Psychiatr Epidemiol 2021; 56(4): 583–92. 32812086 10.1007/s00127-020-01936-3

[9] Sills MR, Shetterly S, Xu S, Magid D, Kempe A. Association between parental depression and children's health care use. Pediatrics 2007; 119(4): e829–36. 17403826 10.1542/peds.2006-2399

[10] Abel KM, Webb RT, Salmon MP, Wan MW, Appleby L. Prevalence and predictors of parenting outcomes in a cohort of mothers with schizophrenia admitted for joint mother and baby psychiatric care in England. J Clin Psychiatry 2005; 66(6): 781–9. 15960575 10.4088/jcp.v66n0618

[11] Webb RT, Pickles AR, Appleby L, Abel KM, Wicks S, Dalman C, Influence of environmental factors in higher risk of sudden infant death syndrome linked with parental mental illness. Arch Gen Psychiatry 2010; 67(1): 69–77. 20048224 10.1001/archgenpsychiatry.2009.172

[12] Minkovitz CS, Strobino D, Scharfstein D, Hou W, Miller T, Mistry KB, Maternal depressive symptoms and children's receipt of health care in the first 3 years of life. Pediatrics 2005; 115: 306–14. 15687437 10.1542/peds.2004-0341

[13] Farr SL, Dietz PM, Rizzo JH, Vesco KK, Callaghan WM, Bruce FC, Health care utilisation in the first year of life among infants of mothers with perinatal depression or anxiety. Paediatr Perinat Epidemiol 2013; 27: 81–8. 23215715 10.1111/ppe.12012 PMC4399813

[14] Raitasalo K, Holmila M, Autti-Rämö I, Notkola IL, Tapanainen H. Hospitalisations and out-of-home placements of children of substance-abusing mothers: a register-based cohort study. Drug Alcohol Rev 2015; 34(1): 38–45. 24602040 10.1111/dar.12121

[15] Sarkola T, Gissler M, Kahila H, Autti-Rämö I, Halmesmäki E. Early healthcare utilization and welfare interventions among children of mothers with alcohol and substance abuse: a retrospective cohort study. Acta Paediatr 2011; 100(10): 1379–85. 21480986 10.1111/j.1651-2227.2011.02317.x

[16] Ranning A, Benros ME, Thorup AAE, Davidsen KA, Hjorthøj C. Morbidity and mortality in the children and young adult offspring of parents with schizophrenia or affective disorders — a nationwide register-based cohort study in 2 million individuals. Schizophr Bull 2020; 46(1): 130–9. 31173637 10.1093/schbul/sbz040 PMC6942150

[17] Dreyer K, Williamson RAP, Hargreaves DS, Rosen R, Deeny SR. Associations between parental mental health and other family factors and healthcare utilisation among children and young people: a retrospective, cross-sectional study of linked healthcare data. BMJ Paediatr Open 2018; 2: e000266. 10.1136/bmjpo-2018-000266 PMC6069921 30094348

[18] Ranning A, Laursen TM, Thorup A, Hjorthøj C, Nordentoft M. Serious mental illness and disrupted caregiving for children: a nationwide, register-based cohort study. J Clin Psychiatry 2015; 76(8): e1006–14. 26335086 10.4088/JCP.13m08931

[19] Wilson C, Hogg R, Henderson M, Wilson P. Patterns of primary care service use by families with young children. Fam Pract 2013; 30(6): 679–94. 24115013 10.1093/fampra/cmt057

[20] Shen H, Magnusson C, Rai D, Lundberg M, Lê-Scherban F, Dalman C, Associations of parental depression with child school performance at age 16 years in Sweden. JAMA Psychiatry 2016; 73(3): 239–46. 26842307 10.1001/jamapsychiatry.2015.2917

[21] Riglin L, Collishaw S, Thapar AK, Dalsgaard S, Langley K, Smith GD, Association of genetic risk variants with attention-deficit/hyperactivity disorder trajectories in the general population. JAMA Psychiatry 2016; 73(12): 1285–92. 27806167 10.1001/jamapsychiatry.2016.2817 PMC6485350

[22] Gilbody S, Peckham E, Bailey D, Arundel C, Heron P, Crosland S, Smoking cessation for people with severe mental illness (SCIMITAR+): a pragmatic randomised controlled trial. Lancet Psychiatry 2019; 6(5): 379–90. 30975539 10.1016/S2215-0366(19)30047-1 PMC6546931

[23] Pierce M, Abel KM, Muwonge Jr J, Wicks S, Nevriana A, Hope H, Prevalence of parental mental illness and association with socioeconomic adversity among children in Sweden between 2006 and 2016: a population-based cohort study. Lancet Public Health 2020; 5(11): e583–91. 33120044 10.1016/S2468-2667(20)30202-4

[24] Abel KM, Heuvelman H, Rai D, Timpson NJ, Sarginson J, Shallcross R, Intelligence in offspring born to women exposed to intimate partner violence: a population-based cohort study. Wellcome Open Res 2019; 4: 107. 31681855 10.12688/wellcomeopenres.15270.1 PMC6820818

[25] NHS Improvement. *Performance of the NHS Provider Sector for the Quarter Ended 30 September 2018*. NHS Improvement, 2018 (https://www.england.nhs.uk/wp-content/uploads/2020/08/performance-of-the-nhs-provider-sector-quarter-4-1819.pdf).

[26] Shribman S, Billingham K. *Healthy Child Programme: Pregnancy and the First Five Years of Life*. Department of Health and Social Care, 2009 (https://www.gov.uk/government/publications/healthy-child-programme-pregnancy-and-the-first-5-years-of-life).

