# Supplementary material for: The healthcare resource impact of maternal mental illness on children and adolescents: UK retrospective cohort study
Source: Br J Psychiatry. 2021 Sep;219(3):515–22. doi: 10.1192/bjp.2021.65 (PMC8387858; doi:10.1192/bjp.2021.65)
Supplement: Supplementary file 1 [file S0007125021000659sup001.docx]

Online appendix

1. List of datasets used in this analysis with key references.
2. eFigure 1 Flow diagram to show how children were selected for the study cohort
3. eTable 1 Description of study cohort during the first year of follow-up
4. eTable 2 Sensitivity analysis of the rate of health use maternal mental illness adjusted for quintile of deprivation
5. eTable 3 Sensitivity analysis of the rate of health use and maternal mental illness (with exposure restricted to 2 years prior to entering an age-group)
6. eTable 4 Rates of primary contacts, prescriptions, referrals, outpatient, hospital admissions and Accident and Emergency visits of children with and without maternal mental illness
7. eTable 5 The rates and rate ratios of prescriptions by type of illness of children with and without maternal mental illness, ordered from highest to lowest proportion of prescriptions
8. eTable 6 Costs and cost differences (CD) of primary contacts, prescriptions, outpatient, hospital admissions and Accident and Emergency visits of children exposed and unexposed to maternal mental illness (MMI)

List of datasets used in this analysis with key references

CPRD primary care dataset

Herrett E, Gallagher AM, Bhaskaran K, Forbes H, Mathur R, van Staa T, et al. Data Resource Profile: Clinical Practice Research Datalink (CPRD). Int J Epidemiol [Internet]. 2015 Jun 1 [cited 2018 May 17];44(3):827–36. Available from: <https://academic.oup.com/ije/article-lookup/doi/10.1093/ije/dyv098>

HES Secondary Care dataset

HES data are collected locally and submitted monthly to be processed into a national dataset. These data are used to plan local resources to meet patient need, so it is important that local healthcare use is reported accurately. HES data handlers have a number of established data quality processes once data is submitted, which include deduplication of events, before releasing the annual national dataset.

[The processing cycle and HES data quality - NHS Digital](https://digital.nhs.uk/data-and-information/data-tools-and-services/data-services/hospital-episode-statistics/the-processing-cycle-and-hes-data-quality)

Index of Multiple Deprivation (IMD)

The English Indices of Deprivation 2010:Summary [Internet]. Government, Communities and Local; 2011 [cited 2019 Jun 12]. Available from: <https://www.gov.uk/government/uploads/system/uploads/attachment_data/file/6871/1871208.pdf>

Cost estimates

Primary Care

Unit Costs of Health and Social Care | PSSRU [Internet]. [cited 2019 Jun 12]. Available from: <https://www.pssru.ac.uk/project-pages/unit-costs/>

Prescriptions

Prescription Cost Analysis - England, 2018 [PAS] - NHS Digital [Internet]. [cited 2019 Jun 12]. Available from: <https://digital.nhs.uk/data-and-information/publications/statistical/prescription-cost-analysis/2018#resources>

NHS reference costs - GOV.UK [Internet]. [cited 2019 Jun 12]. Available from: <https://www.gov.uk/government/collections/nhs-reference-costs>

Inflation adjusment

Consumer price inflation time series - Office for National Statistics [Internet]. [cited 2019 Jul 17]. Available from: <https://www.ons.gov.uk/economy/inflationandpriceindices/datasets/consumerpriceindices>

Population estimates

Office of National Statistics. Estimates of the population for the UK, England and Wales, Scotland and Northern Ireland - Office for National Statistics [Internet]. [cited 2019 Jun 3]. Available from: <https://www.ons.gov.uk/peoplepopulationandcommunity/populationandmigration/populationestimates/datasets/populationestimatesforukenglandandwalesscotlandandnorthernireland>


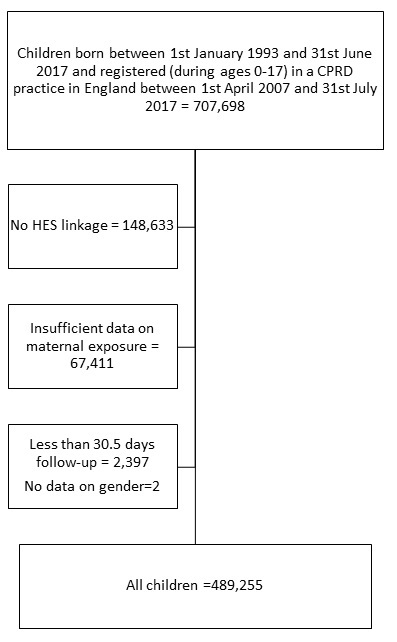


eFigure 1 Flow diagram to show how children were selected for the study cohort

eTable 1 Description of study cohort during the first year of follow-up

| **Characteristics** | **Unexposed** (376,514) | | **Exposed (**112,741) | |
| --- | --- | --- | --- | --- |
|  | N | % | N | % |
| **Age groups*** | |  |  |  |
| under 1 | 200,310 | 80·8 | 47,711 | 19·2 |
| 1 to 4 | 231,966 | 72·8 | 86,497 | 27·2 |
| 5 to 9 | 182,524 | 70·6 | 76,080 | 29·4 |
| 10 to 13 | 126,399 | 70·6 | 52,573 | 29·4 |
| 14 to 17 | 73,107 | 69·0 | 32,865 | 31·0 |
| **Gender** |  |  |  |  |
| Girls | 183,589 | 48·8 | 54,517 | 48·7 |
| Boys | 192,925 | 51·2 | 58,224 | 51·3 |
| **IMD quintile** | |  |  |  |
| Least 1 | 86,431 | 23·0 | 19,293 | 17·1 |
| 2 | 75,110 | 20·0 | 19,685 | 17·5 |
| 3 | 65,823 | 17·5 | 19,956 | 17·7 |
| 4 | 44,188 | 17·4 | 22,456 | 19·9 |
| Most 5 | 56,422 | 15·0 | 22,187 | 19·7 |
| Missing | 27,054 | 7·2 | 9,164 | 8·1 |
| **Region** |  |  |  |  |
| East Midlands | 11,364 | 3·02 | 4,417 | 3·92 |
| East of England | 46,411 | 12·3 | 13,212 | 12·0 |
| London | 56,157 | 14·9 | 9,684 | 8·6 |
| North East | 8,659 | 2·3 | 3,241 | 2·9 |
| North West | 53,383 | 14·2 | 19,745 | 17·5 |
| South Central | 51,157 | 13·6 | 16,357 | 14·5 |
| South East Coast | 49,188 | 13·1 | 13,279 | 11·8 |
| South West | 44,340 | 11·8 | 15,546 | 13·8 |
| West Midlands | 42,172 | 11·2 | 13,027 | 11·6 |
| Yorkshire & The Humber | 13,683 | 3·6 | 4,233 | 3·8 |
| **Ethnicity** |  |  |  |  |
| Asian/British Asian | 21,307 | 5·7 | 2,321 | 2·1 |
| Black/ black British | 11,497 | 3·1 | 1192 | 1·1 |
| Mixed | 11,051 | 2·9 | 2,831 | 2·5 |
| Other | 6,137 | 1·6 | 991 | 0·9 |
| White | 283,183 | 75·2 | 93,168 | 82·6 |
| Unknown | 18,103 | 4·8 | 4,673 | 4·1 |
| Missing | 25,236 | 6·7 | 7,565 | 6·7 |
| Median (IQR) age of mother at child's birth | 31 | (27-34) | 30 | (25-34) |
|  |  |  |  |  |
| Median (IQR) length of follow-up(years) | 5·0 | (2·7-7·5) | 5·3 | (2·9-7·7) |

*children appear in more than one age-group

eTable 2 Sensitivity analysis of the rate of health use maternal mental illness adjusted for quintile of IMD

|  |  | Exposed |  |  |  |  | Unexposed |
| --- | --- | --- | --- | --- | --- | --- | --- |
|  | N | rate | RR | 95% CI | RD | 95% CI | Rate |
| Overall | 453,037 | 12·0 | 1·26 | (1·24-1·27) | 2·16 | (2·08-2·24) | 9·87 |
|  |  |  |  |  |  |  |  |
| under 1 | 247,872 | 22·0 | 1·20 | (1·19-1·21) | 3·66 | (3·46-3·85) | 18·3 |
| 1 to 4 | 318,283 | 11·2 | 1·18 | (1·17-1·19) | 1·72 | (1·62-1·82) | 9·5 |
| 5 to 9 | 258,461 | 7·2 | 1·27 | (1·25-1·29) | 1·53 | (1·43-1·62) | 5·6 |
| 10 to 13 | 147,104 | 7·0 | 1·32 | (1·30-1·35) | 1·71 | (1·58-1·83) | 5·3 |
| 14 to 17 | 71,542 | 8·7 | 1·31 | (1·28-1·34) | 2·06 | (1·86-2·25) | 6·6 |

eTable 3 Sensitivity analysis of the rate of health use and maternal mental illness (with exposure restricted to 2 years prior to entering an age-group)

|  |  | Exposed |  |  |  |  | Unexposed |
| --- | --- | --- | --- | --- | --- | --- | --- |
|  | N | rate | RR | 95% CI | RD | 95% CI | Rate |
| All | 459,269 | 11·3 | 1·26 | (1·25-1·27) | 2·08 | (1·99-2·17) | 9·19 |
|  |  |  |  |  |  |  |  |
| under 1 | 178,771 | 22·5 | 1·19 | (1·17-1·20) | 3·47 | (3·22-3·72) | 19·1 |
| 1 to 4 | 253,224 | 11·6 | 1·18 | (1·17-1·19) | 1·73 | (1·60-1·86) | 9·85 |
| 5 to 9 | 249,753 | 7·54 | 1·29 | (1·27-1·31) | 1·65 | (1·54-1·76) | 5·79 |
| 10 to 13 | 173,672 | 7·14 | 1·33 | (1·30-1·35) | 1·75 | (1·62-1·89) | 5·33 |
| 14 to 17 | 103,368 | 8·93 | 1·31 | (1·29-1·34) | 2·06 | (1·88-2·24) | 6·66 |

eTable 4 Sensitivity analysis of the rate of primary care contacts and maternal mental illness for children without linkage to Hospital Episodes Data (HES)

|  |  | Exposed |  |  |  |  | Unexposed |
| --- | --- | --- | --- | --- | --- | --- | --- |
|  | N | rate | RR | 95% CI | RD | 95% CI | Rate |
| All | 630,351 | 4.83 | 1·25 | (1·24-1·25) | 0.75 | (0.73-0.77) | 4.08 |
|  |  |  |  |  |  |  |  |
| under 1 | 328,885 | 10.6 | 1·14 | (1·13-1·15) | 1.34 | (1.28-1.41) | 9.21 |
| 1 to 4 | 415,501 | 4.59 | 1·17 | (1·16-1·17) | 0.65 | (0.63-0.68) | 3.93 |
| 5 to 9 | 339,233 | 2.26 | 1·27 | (1·26-1·28) | 0.48 | (0.46-0.49) | 1.78 |
| 10 to 13 | 235,244 | 2.10 | 1·34 | (1·32-1·35) | 0.52 | (0.50-0.54) | 1.55 |
| 14 to 17 | 137,115 | 2.82 | 1·33 | (1·31-1·34) | 0.70 | (0.66-0.73) | 2.13 |

eTable 5 Rates, rate ratios (RR) and rate differences (RD) of primary contacts, prescriptions, referrals, outpatient, hospital admissions and Accident and Emergency visits of children exposed and unexposed to maternal mental illness·

| ` | **Common** | |  |  |  | **Serious** |  |  |  |  | **Addict** | |  |  |  | **Any** |  |  |  |  | **Ref** |
| --- | --- | --- | --- | --- | --- | --- | --- | --- | --- | --- | --- | --- | --- | --- | --- | --- | --- | --- | --- | --- | --- |
|  | **rate** | RR | 95% CI | RD | 95% CI | **rate** | RR | 95% CI | RD | 95% CI | **rate** | RR | 95% CI | RD | 95% CI | **rate** | RR | 95% CI | RD | 95% CI | **Rate*** |
| Primary contacts | | |  |  |  |  |  |  |  |  |  |  |  |  |  |  |  |  |  |  |  |
| Overall | **5·03** | 1·24 | 1·23-1·25 | 0·76 | 0·73-0·78 | **4·66** | 1·31 | 1·26-1·35 | 0·67 | 0·50-0·83 | **4·71** | 1·15 | 1·11-1·18 | 0·44 | 0·29-0·58 | **5·02** | 1·24 | 1·23-1·23 | 0·75 | 0·73-0·78 | **4·27** |
| under 1 | **11·1** | 1·14 | 1·14-1·15 | 1·39 | 1·31-1·46 | **10·1** | 1·04 | 0·99-1·09 | 0·39 | -0·06-0·85 | **10·63** | 1·09 | 1·05-1·14 | 0·92 | 0·47-1·36 | **11·1** | 1·14 | 1·13-1·15 | 1·37 | 1·30-1·44 | **9·72** |
| 1 to 4 | **4·78** | 1·15 | 1·15-1·16 | 0·64 | 0·61-0·67 | **4·72** | 1·14 | 1·09-1·19 | 0·58 | 0·38-0·78 | **4·29** | 1·04 | 1·00-1·08 | 0·15 | -0·01-0·32 | **4·77** | 1·15 | 1·14-1·16 | 0·63 | 0·60-0·66 | **4·14** |
| 5 to 9 | **2·34** | 1·26 | 1·24-1·27 | 0·48 | 0·46-0·50 | **2·66** | 1·42 | 1·34-1·51 | 0·79 | 0·64-0·94 | **2·16** | 1·16 | 1·10-1·22 | 0·30 | 0·19-0·40 | **2·34** | 1·25 | 1·24-1·27 | 0·47 | 0·45-0·50 | **1·87** |
| 10 to 13 | **2·15** | 1·33 | 1·31-1·35 | 0·53 | 0·51-0·56 | **2·45** | 1·51 | 1·41-1·62 | 0·83 | 0·66-1·00 | **1·97** | 1·21 | 1·14-1·29 | 0·35 | 0·23-0·47 | **2·15** | 1·33 | 1·31-1·34 | 0·53 | 0·51-0·56 | **1·62** |
| 14 to 17 | **2·88** | 1·32 | 1·31-1·34 | 0·70 | 0·67-0·74 | **3·25** | 1·50 | 1·38-1·63 | 1·08 | 0·81-1·35 | **2·68** | 1·24 | 1·16-1·32 | 0·51 | 0·33-0·69 | **2·87** | 1·32 | 1·30-1·34 | 0·70 | 0·66-0·74 | **2·17** |
| Referrals |  |  |  |  |  |  |  |  |  |  |  |  |  |  |  |  |  |  |  |  |  |
| Overall | **0·21** | 1·30 | 1·29-1·32 | 0·04 | 0·04-0·05 | **0·22** | 1·41 | 1·33-1·50 | 0·05 | 0·07-0·07 | **0·21** | 1·16 | 1·10-1·23 | 0·02 | 0·01-0·03 | **0·21** | 1·30 | 1·29-1·31 | 0·04 | 0·04-0·05 | **0·16** |
| under 1 | **0·33** | 1·20 | 1·17-1·23 | 0·05 | 0·05-0·06 | **0·32** | 1·19 | 1·02-1·39 | 0·05 | 0·10-0·10 | **0·29** | 1·07 | 0·91-1·26 | 0·02 | -0·03-0·07 | **0·32** | 1·20 | 1·17-1·23 | 0·05 | 0·05-0·05 | **0·27** |
| 1 to 4 | **0·18** | 1·25 | 1·23-1·27 | 0·03 | 0·03-0·04 | **0·17** | 1·23 | 1·11-1·35 | 0·03 | 0·05-0·05 | **0·15** | 1·05 | 0·95-1·16 | 0·01 | -0·01-0·02 | **0·18** | 1·24 | 1·22-1·26 | 0·03 | 0·03-0·03 | **0·14** |
| 5 to 9 | **0·16** | 1·37 | 1·34-1·39 | 0·04 | 0·04-0·04 | **0·19** | 1·64 | 1·48-1·81 | 0·07 | 0·09-0·09 | **0·15** | 1·32 | 1·21-1·43 | 0·04 | 0·02-0·05 | **0·16** | 1·36 | 1·34-1·39 | 0·04 | 0·04-0·04 | **0·11** |
| 10 to 13 | **0·16** | 1·37 | 1·34-1·40 | 0·04 | 0·04-0·05 | **0·17** | 1·47 | 1·31-1·65 | 0·05 | 0·07-0·07 | **0·14** | 1·21 | 1·08-1·35 | 0·02 | 0·01-0·04 | **0·16** | 1·37 | 1·34-1·40 | 0·04 | 0·04-0·04 | **0·11** |
| 14 to 17 | **0·21** | 1·34 | 1·31-1·37 | 0·05 | 0·05-0·06 | **0·25** | 1·59 | 1·39-1·82 | 0·09 | 0·13-0·13 | **0·18** | 1·17 | 1·04-1·32 | 0·03 | 0·01-0·05 | **0·21** | 1·34 | 1·31-1·37 | 0·05 | 0·05-0·05 | **0·16** |
| Prescriptions | |  |  |  |  |  |  |  |  |  |  |  |  |  |  |  |  |  |  |  |  |
| Overall | **4·93** | 1·28 | 1·26-1·30 | 1·03 | 0·98-1·08 | **5·22** | 1·42 | 1·33-1·51 | 1·32 | 0·98-1·67 | **4·75** | 1·19 | 1·12-1·27 | 0·85 | 0·54-1·15 | **4·92** | 1·28 | 1·26-1·29 | 1·02 | 0·97-1·07 | **3·90** |
| under 1 | **8·23** | 1·29 | 1·27-1·30 | 1·83 | 1·71-1·94 | **7·71** | 1·20 | 1·08-1·34 | 1·30 | 0·47-2·14 | **8·23** | 1·28 | 1·17-1·42 | 1·82 | 1·03-2·62 | **8·22** | 1·28 | 1·26-1·30 | 1·81 | 1·70-1·93 | **6·40** |
| 1 to 4 | **4·60** | 1·19 | 1·17-1·20 | 0·72 | 0·66-0·78 | **4·89** | 1·26 | 1·16-1·37 | 1·01 | 0·60-1·43 | **4·40** | 1·14 | 1·04-1·24 | 0·53 | 0·15-0·90 | **4·60** | 1·19 | 1·17-1·20 | 0·72 | 0·66-0·78 | **3·88** |
| 5 to 9 | **3·37** | 1·27 | 1·24-1·29 | 0·71 | 0·64-0·77 | **4·08** | 1·53 | 1·37-1·71 | 1·42 | 0·97-1·87 | **3·30** | 1·24 | 1·11-1·39 | 0·64 | 0·26-1·01 | **3·37** | 1·27 | 1·24-1·29 | 0·71 | 0·64-0·77 | **2·66** |
| 10 to 13 | **3·52** | 1·35 | 1·31-1·38 | 0·91 | 0·82-1·00 | **3·95** | 1·51 | 1·34-1·71 | 1·34 | 0·86-1·82 | **3·01** | 1·15 | 1·02-1·29 | 0·39 | 0·04-0·75 | **3·51** | 1·34 | 1·31-1·38 | 0·90 | 0·81-0·98 | **2·61** |
| 14 to 17 | **4·34** | 1·32 | 1·28-1·36 | 1·05 | 0·93-1·16 | **5·36** | 1·63 | 1·43-1·85 | 2·06 | 1·37-2·75 | **3·80** | 1·15 | 1·01-1·31 | 0·50 | 0·02-0·99 | **4·33** | 1·31 | 1·28-1·35 | 1·04 | 0·92-1·15 | **3·30** |
| Outpatient visits | | |  |  |  |  |  |  |  |  |  |  |  |  |  |  |  |  |  |  |  |
| Overall | **0·99** | 1·30 | 1·28-1·32 | 0·23 | 0·22-0·24 | **1·07** | 1·41 | 1·30-1·53 | 0·31 | 0·22-0·39 | **1·09** | 1·32 | 1·23-1·42 | 0·32 | 0·24-0·40 | **0·99** | 1·30 | 1·28-1·32 | 0·23 | 0·22-0·24 | **0·76** |
| under 1 | **1·37** | 1·22 | 1·19-1·25 | 0·25 | 0·22-0·28 | **1·42** | 1·26 | 1·08-1·47 | 0·29 | 0·07-0·52 | **1·60** | 1·42 | 1·24-1·62 | 0·47 | 0·26-0·68 | **1·37** | 1·22 | 1·19-1·25 | 0·25 | 0·22-0·28 | **1·13** |
| 1 to 4 | **0·99** | 1·34 | 1·31-1·37 | 0·25 | 0·23-0·27 | **1·08** | 1·45 | 1·30-1·62 | 0·34 | 0·22-0·46 | **1·15** | 1·55 | 1·40-1·72 | 0·41 | 0·29-0·53 | **0·99** | 1·34 | 1·31-1·37 | 0·25 | 0·23-0·27 | **0·74** |
| 5 to 9 | **0·93** | 1·36 | 1·33-1·39 | 0·25 | 0·23-0·26 | **1·03** | 1·50 | 1·34-1·68 | 0·34 | 0·23-0·46 | **1·00** | 1·45 | 1·31-1·61 | 0·31 | 0·21-0·41 | **0·93** | 1·36 | 1·33-1·39 | 0·25 | 0·23-0·26 | **0·68** |
| 10 to 13 | **0·76** | 1·37 | 1·33-1·41 | 0·20 | 0·19-0·22 | **0·87** | 1·56 | 1·35-1·81 | 0·31 | 0·19-0·44 | **0·70** | 1·27 | 1·10-1·45 | 0·15 | 0·05-0·24 | **0·76** | 1·37 | 1·33-1·40 | 0·20 | 0·18-0·22 | **0·55** |
| 14 to 17 | **0·65** | 1·22 | 1·17-1·27 | 0·12 | 0·09-0·14 | **0·69** | 1·29 | 1·05-1·58 | 0·15 | 0·01-0·29 | **0·53** | 0·99 | 0·81-1·23 | 0·00 | -0·12-0·11 | **0·65** | 1·22 | 1·17-1·27 | 0·12 | 0·09-0·14 | **0·54** |
| Hospital admissions | | |  |  |  |  |  |  |  |  |  |  |  |  |  |  |  |  |  |  |  |
| Overall | **0·21** | 1·37 | 1·32-1·42 | 0·06 | 0·06-0·07 | **0·14** | 1·38 | 1·22-1·56 | 0·01 | 0·10-0·00 | **0·21** | 1·46 | 1·27-1·69 | 0·07 | 0·04-0·10 | **0·21** | 1·37 | 1·32-1·42 | 0·06 | 0·06-0·07 | **0·14** |
| under 1 | **0·45** | 1·52 | 1·48-1·57 | 0·16 | 0·14-0·17 | **0·30** | 1·52 | 1·24-1·86 | 0·17 | 0·00-0·06 | **0·46** | 1·54 | 1·29-1·84 | 0·16 | 0·08-0·25 | **0·46** | 1·52 | 1·48-1·57 | 0·16 | 0·14-0·17 | **0·30** |
| 1 to 4 | **0·20** | 1·39 | 1·33-1·44 | 0·06 | 0·05-0·06 | **0·14** | 1·41 | 1·21-1·64 | 0·05 | 0·00-0·03 | **0·21** | 1·47 | 1·20-1·81 | 0·07 | 0·03-0·11 | **0·20** | 1·39 | 1·33-1·44 | 0·06 | 0·05-0·06 | **0·15** |
| 5 to 9 | **0·11** | 1·35 | 1·27-1·43 | 0·03 | 0·02-0·03 | **0·08** | 1·54 | 1·26-1·89 | 0·04 | 0·00-0·02 | **0·10** | 1·32 | 1·14-1·53 | 0·03 | 0·01-0·04 | **0·11** | 1·35 | 1·27-1·43 | 0·03 | 0·02-0·03 | **0·08** |
| 10 to 13 | **0·08** | 1·36 | 1·26-1·47 | 0·02 | 0·02-0·03 | **0·06** | 1·33 | 1·07-1·65 | 0·02 | 0·02-0·00 | **0·08** | 1·33 | 1·05-1·69 | 0·02 | 0·00-0·04 | **0·08** | 1·36 | 1·26-1·47 | 0·02 | 0·02-0·03 | **0·06** |
| 14 to 17 | **0·06** | 1·24 | 1·11-1·39 | 0·01 | 0·01-0·02 | **0·05** | 1·15 | 0·82-1·60 | 0·00 | 0·44--0·01 | **0·08** | 1·69 | 0·97-2·93 | 0·03 | -0·01-0·08 | **0·06** | 1·24 | 1·11-1·38 | 0·01 | 0·01-0·02 | **0·05** |
| Accident and Emergency | | | |  |  |  |  |  |  |  |  |  |  |  |  |  |  |  |  |  |  |
| Overall | **0·43** | 1·34 | 1·33-1·36 | 0·11 | 0·11-0·11 | **0·41** | 1·30 | 1·23-1·39 | 0·09 | 0·07-0·12 | **0·43** | 1·31 | 1·25-1·38 | 0·11 | 0·08-0·14 | **0·43** | 1·34 | 1·32-1·35 | 0·11 | 0·11-0·11 | **0·32** |
| under 1 | **0·65** | 1·38 | 1·35-1·41 | 0·18 | 0·17-0·19 | **0·56** | 1·19 | 1·04-1·36 | 0·09 | 0·01-0·17 | **0·68** | 1·46 | 1·27-1·66 | 0·21 | 0·12-0·31 | **0·65** | 1·38 | 1·35-1·41 | 0·18 | 0·17-0·19 | **0·47** |
| 1 to 4 | **0·51** | 1·30 | 1·28-1·32 | 0·12 | 0·11-0·12 | **0·51** | 1·31 | 1·20-1·42 | 0·12 | 0·08-0·16 | **0·49** | 1·26 | 1·17-1·36 | 0·10 | 0·07-0·14 | **0·51** | 1·30 | 1·28-1·32 | 0·12 | 0·11-0·12 | **0·39** |
| 5 to 9 | **0·30** | 1·33 | 1·31-1·35 | 0·07 | 0·07-0·08 | **0·33** | 1·46 | 1·33-1·60 | 0·10 | 0·07-0·13 | **0·30** | 1·31 | 1·21-1·42 | 0·07 | 0·05-0·09 | **0·30** | 1·33 | 1·31-1·35 | 0·07 | 0·07-0·08 | **0·23** |
| 10 to 13 | **0·34** | 1·36 | 1·33-1·39 | 0·09 | 0·08-0·10 | **0·32** | 1·28 | 1·15-1·42 | 0·07 | 0·03-0·10 | **0·31** | 1·27 | 1·17-1·39 | 0·07 | 0·04-0·10 | **0·34** | 1·36 | 1·33-1·39 | 0·09 | 0·08-0·09 | **0·25** |
| 14 to 17 | **0·21** | 1·33 | 1·29-1·37 | 0·05 | 0·05-0·06 | **0·21** | 1·30 | 1·10-1·53 | 0·05 | 0·01-0·08 | **0·20** | 1·28 | 1·12-1·46 | 0·04 | 0·02-0·07 | **0·21** | 1·33 | 1·29-1·37 | 0·05 | 0·05-0·06 | **0·16** |

*children unexposed to maternal mental illness

eTable 6 The rates and rate ratios of prescriptions by type of illness of children (0-17 years) with and without maternal mental illness, ordered from highest to lowest proportion of total child prescriptions

| Drug purpose (BNF chapter) |  | Rate (per person-year) | |  |  |
| --- | --- | --- | --- | --- | --- |
|  | % of meds | Any MMI | Unexposed | RR | (95% CI) |
| Skin (e.g. eczema, psoriasis, acne) | 25.0 | 1.06 | 1.06 | 1.00 | (1.00-1.01) |
| Respiratory system (e.g. asthma and allergies) | 17.6 | 0.72 | 0.54 | 1.35 | (1.34-1.36) |
| Infections (e.g. antibiotics) | 16.7 | 0.78 | 0.62 | 1.25 | (1.25-1.26) |
| Central Nervous system (e.g. Mental and behavioural disorders and epilepsy) | 7.6 | 0.43 | 0.25 | 1.77 | (1.76-1.79) |
| Gastro-intestinal system (e.g. dyspepsia, diarrhoea, chronic bowel disorders) | 6.6 | 0.49 | 0.30 | 1.54 | (1.53-1.55) |
| Nutrition and blood (e.g. anaemia and vitamin deficiency) | 6.3 | 0.51 | 0.34 | 1.23 | (1.22-1.24) |
| Eye (e.g. antibiotics and anti-inflammatories for the eye) | 4.0 | 0.21 | 0.19 | 1.15 | (1.14-1.16) |
| Ear, nose and throat (e.g. otitis, nasal allergies, throat ulceration) | 3.0 | 0.15 | 0.13 | 1.20 | (1.19-1.21) |
| Endocrine system (e.g. type 1 diabetes and metabolic disorders) | 2.4 | 0.08 | 0.07 | 1.19 | (1.17-1.22) |
| Musculoskeletal disease (e.g. juvenile arthritis) | 1.8 | 0.08 | 0.06 | 1.45 | (1.44-1.47) |
| Obstetrics, gynaecology and urinary tract disorders (e.g enuresis, contraception) | 1.2 | 0.06 | 0.05 | 1.41 | (1.39-1.43) |

BNF chapter Immunisations were excluded because this information is contained elsewhere.

BNF chapters for Cardiovascular disease, Anaesthetics and Malignant disease were excluded because they contribute <1% to prescription total.

| **Healthcare type** | **Exposed to MI** | **Unexposed to MMI** | **CD (per child per year)** | **95% CI** | **Total CD** | **CD/ Total CD** |
| --- | --- | --- | --- | --- | --- | --- |
| **Total** | |  |  |  |  |  |
| under 1 | £3,076 | £2,211 | £864 | £ 810 - £ 918 |  | **-** |
| 1 to 4 | £1,128 | £875 | £253 | £ 234 - £ 272 |  | - |
| 5 to 9 | £764 | £616 | £148 | £ 133 - £ 163 |  | - |
| 10 to 13 | £745 | £572 | £174 | £ 156 - £ 192 |  | - |
| 14 to 17 | £618 | £494 | £124 | £ 104 - £ 143 |  | - |
| **Primary care** | |  |  |  |  |  |
| under 1 | £292 | £248 | £44 | £ 9 - £ 10 | £864 | 0·05 |
| 1 to 4 | £124 | £106 | £19 | £ 42 - £ 46 | £253 | 0·07 |
| 5 to 9 | £67 | £54 | £13 | £ 18 - £ 19 | £148 | 0·09 |
| 10 to 13 | £62 | £46 | £16 | £ 13 - £ 14 | £174 | 0·09 |
| 14 to 17 | £82 | £62 | £20 | £ 15 - £ 16 | £124 | 0·16 |
| **Prescriptions** | |  |  |  |  |  |
| under 1 | £82 | £60 | £22 | £ 21 - £ 24 | £864 | 0·03 |
| 1 to 4 | £44 | £36 | £9 | £ 8 - £ 10 | £253 | 0·03 |
| 5 to 9 | £42 | £32 | £10 | £ 9 - £ 11 | £148 | 0·07 |
| 10 to 13 | £51 | £36 | £15 | £ 13 - £ 16 | £174 | 0·08 |
| 14 to 17 | £55 | £42 | £13 | £ 10 - £ 15 | £124 | 0·10 |
| **Outpatients** | |  |  |  |  |  |
| under 1 | £1,161 | £902 | £259 | £ 236 - £ 283 | £864 | 0·30 |
| 1 to 4 | £387 | £314 | £73 | £ 67 - £ 79 | £253 | 0·29 |
| 5 to 9 | £383 | £327 | £56 | £ 50 - £ 62 | £148 | 0·38 |
| 10 to 13 | £390 | £316 | £74 | £ 66 - £ 82 | £174 | 0·43 |
| 14 to 17 | £286 | £247 | £39 | £ 30 - £ 48 | £124 | 0·31 |
| **Hospital admissions** | |  |  |  |  |  |
| under 1 | £1,448 | £935 | £513 | £ 64 - £ 80 | £864 | 0·59 |
| 1 to 4 | £507 | £368 | £139 | £ 473 - £ 553 | £253 | 0·55 |
| 5 to 9 | £233 | £173 | £60 | £ 124 - £ 154 | £148 | 0·41 |
| 10 to 13 | £197 | £140 | £58 | £ 49 - £ 71 | £174 | 0·33 |
| 14 to 17 | £166 | £121 | £45 | £ 45 - £ 70 | £124 | 0·36 |
| **A&E** |  |  |  |  |  |  |
| under 1 | £93 | £66 | £26 | £ 24 - £ 28 | £864 | 0·03 |
| 1 to 4 | £66 | £51 | £14 | £ 14 - £ 15 | £253 | 0·06 |
| 5 to 9 | £38 | £29 | £9 | £ 8 - £ 10 | £148 | 0·06 |
| 10 to 13 | £46 | £34 | £12 | £ 11 - £ 13 | £174 | 0·07 |
| 14 to 17 | £30 | £23 | £7 | £ 6 - £ 8 | £124 | 0·06 |

eTable 7 Costs and cost differences (CD) of primary contacts, prescriptions, outpatient, hospital admissions and Accident and Emergency visits of children exposed and unexposed to maternal mental illness (MMI)
